# TECHNOLOGY-BASED PHYSIO-FEEDBACK EXERCISE PROGRAM (PEER) TO PREVENT FALLS AND ENHANCE HEALTH EQUITY

**DOI:** 10.1093/geroni/igae098.2119

**Published:** 2024-12-31

**Authors:** Ladda Thiamwong, Rui Xie, Nichole Lighthall, Victoria Loerzel, Joon-Hyuk Park, Jeffrey Stout

**Affiliations:** University of Central Florida, Orlando, Florida, United States; University of Central Florida, Orlando, Florida, United States; University of Central Florida, Orlando, Florida, United States; University of Central Florida, Orlando, Florida, United States; University of Central Florida, Orlando, Florida, United States; University of Central Florida, Orlando, Florida, United States

## Abstract

Only a few studies have focused on low-income older adults with a mismatch between perceived fall risk and physiological fall risk. We developed a Physio-fEedback Exercise pRogram (PEER) which includes a) physio-feedback using a real-time portable innovative technology—the BTrackS Balance System: BBS; b) cognitive reframing based on a novel fall risk appraisal matrix; and c) peer-led exercise by focusing on balance and strength training. This NIH-funded clustered randomized control trial is led by an interdisciplinary team representing nursing, kinesiology, cognitive psychology, engineering, and data science. We aim to prevent falls and reduce health disparities by facilitating shifts from maladaptive to adaptive fall risk appraisal (i.e., moving from irrational, incongruent, or congruent to the rational quadrant) The 8-week PEER intervention is offered at low-income independent living communities/units in Central Florida through our partnership with community-based and public health organizations. We collected data at baseline and measured outcomes after program completion and found that 17.78 % of the participants in the PEER group (n=45) had adaptive shifting compared to 13.46 % in the control group (n=52). Up to 24.99% of the control group had maladaptive shifting compared to only 12.73% of the PEER group. The PEER intervention results in a shift from maladaptive to adaptive as a way of preventing falls, which is novel, and PEER may slow down the maladaptive shifting process. Integrating technology into fall risk assessments could increase engagement in fall interventions in low-income settings due to barriers faced by older adults regarding accessibility, knowledge, and healthcare costs.

